# Prelude to Passion: Limbic Activation by “Unseen” Drug and Sexual Cues

**DOI:** 10.1371/journal.pone.0001506

**Published:** 2008-01-30

**Authors:** Anna Rose Childress, Ronald N. Ehrman, Ze Wang, Yin Li, Nathan Sciortino, Jonathan Hakun, William Jens, Jesse Suh, John Listerud, Kathleen Marquez, Teresa Franklin, Daniel Langleben, John Detre, Charles P. O'Brien

**Affiliations:** 1 Department of Psychiatry, University of Pennsylvania School of Medicine, Philadelphia, Pennsylvania, United States of America; 2 Department of Neurology, University of Pennsylvania School of Medicine, Philadelphia, Pennsylvania, United States of America; 3 Veterans Integrated Services Network (VISN) 4, Mental Illness Reasearch, Education and Clinical Centers (MIRECC), Philadelphia Department of Veterans Affairs Medical Center, Philadelphia, Pennsylvania, United States of America; University of Minnesota, United States of America

## Abstract

**Background:**

The human brain responds to recognizable signals for sex and for rewarding drugs of abuse by activation of limbic reward circuitry. Does the brain respond in similar way to such reward signals even when they are “unseen”, i.e., presented in a way that prevents their conscious recognition? Can the brain response to “unseen” reward cues predict the future affective response to recognizable versions of such cues, revealing a link between affective/motivational processes inside and outside awareness?

**Methodology/Principal Findings:**

We exploited the fast temporal resolution of event-related functional magnetic resonance imaging (fMRI) to test the brain response to “unseen” (backward-masked) cocaine, sexual, aversive and neutral cues of 33 milliseconds duration in male cocaine patients (n = 22). Two days after scanning, the affective valence for visible versions of each cue type was determined using an affective bias (priming) task. We demonstrate, for the first time, limbic brain activation by “unseen” drug and sexual cues of only 33 msec duration. Importantly, increased activity in an large interconnected ventral pallidum/amygdala cluster to the “unseen” cocaine cues strongly predicted future positive affect to visible versions of the same cues in subsequent off-magnet testing, pointing both to the functional significance of the rapid brain response, and to shared brain substrates for appetitive motivation within and outside awareness.

**Conclusions/Significance:**

These findings represent the first evidence that brain reward circuitry responds to drug and sexual cues presented outside awareness. The results underscore the sensitivity of the brain to “unseen” reward signals and may represent the brain's primordial signature for desire. The limbic brain response to reward cues outside awareness may represent a potential vulnerability in disorders (e.g., the addictions) for whom poorly-controlled appetitive motivation is a central feature.

## Introduction

At the end of the nineteenth century, Freud[1] proposed that much of human motivation – both fears and desires – occurs outside awareness. This notion had a profound impact on the culture of the past century, without being easily testable. Functional brain imaging can now be used to test whether the brain responds to cues of motivational significance, even when presented outside awareness. Neuroimaging studies have shown that the brain (e.g., the amygdala) can respond to very brief signals for fear or threat, even when presented by procedures that prevent conscious recognition [2]–[6]. Hair-trigger responses to learned threat cues, even “unseen” threat, may have been shaped by their survival advantage.

Some reward signals (e.g., cues for sexual opportunity[7]) also have clear survival significance; others (e.g., learned cues for drug rewards[8]) have powerful motivational effects by their actions on natural reward circuits, but confer no survival advantage. Whether the brain responds to reward signals of either type – when presented outside awareness – is not yet known; we tested both types in the current study.

**Figure 1:**
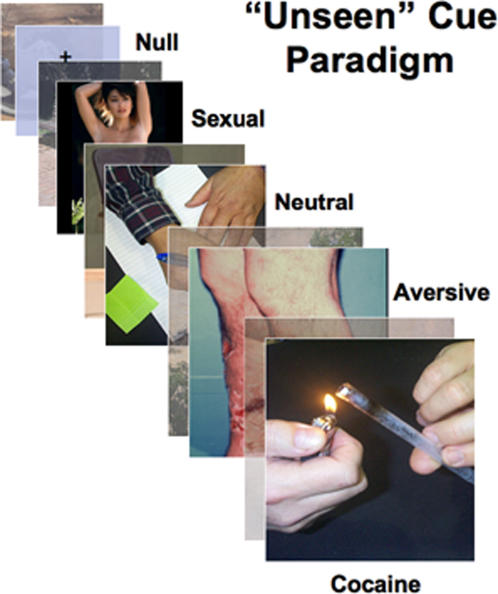
“Unseen” cue paradigm. 24 randomly-presented 33 msec targets in each of four categories (cocaine, sexual, aversive and neutral, interspersed with grey-screen nulls) were immediately followed by a 467 msec neutral “masking” stimulus”. Under these conditions, the 33 msec stimuli can escape conscious detection (see Methods for additional task details).

Extended – seconds or minutes-long – exposures to relevant cocaine[9] and sexual[10], [11] stimuli can trigger conscious desire and activation of interconnected limbic (e.g., amygdala, striatum/pallidum, orbitofrontal cortex, temporal pole, insula and medial prefrontal cortex) brain regions important in reward learning[12]. To investigate the possibility of a similar brain response pattern to such cues when presented outside awareness, we made use of visual “backward masking” (see Methods), in which very brief targets are each immediately followed by much longer stimuli of different content. Under these conditions, subjects report having seen the longer stimuli, but the brief targets escape visual recognition and are reported as “unseen” (see Methods). For our sample of 22 male cocaine patients (average age 41; average 15.7 years of cocaine use), the target stimuli were randomly-presented cocaine-related, sexual, aversive, and neutral visual cues of 33 milliseconds duration, each followed by neutral picture masks of 467 milliseconds duration (Figure 1, below). We used fast event-related fMRI to enable rapid (average inter-stimulus 2 seconds) presentation of 24 unique stimuli in each category, along with 24 interspersed presentations of a null (grey screen with fixation cross). Both an *immediate recall task* in the imaged patients and a *forced choice categorization task* in an independent sample (see Methods) were used to assess adequacy of the masking procedure. Two days later (off-magnet), we tested a subgroup of the imaging cohort with a priming task (see Methods) to determine the affective (positive or negative) valence of the same targets when clearly visible.

## Results

### Backward-masking

Results from both the immediate recall task and the forced-choice categorization recognition task indicated that the backward-masking parameters were effective in preventing recognition of the targets (see details in supplemental Results in Text S1; Table S1). The subjective debriefing following each task was consistent with the objective (immediate recall or forced-choice categorization) task data; the target stimuli were not “seen” under the temporal parameters of the study.

### Limbic activation by “unseen” cocaine and sexual cues

**Figure 2:**
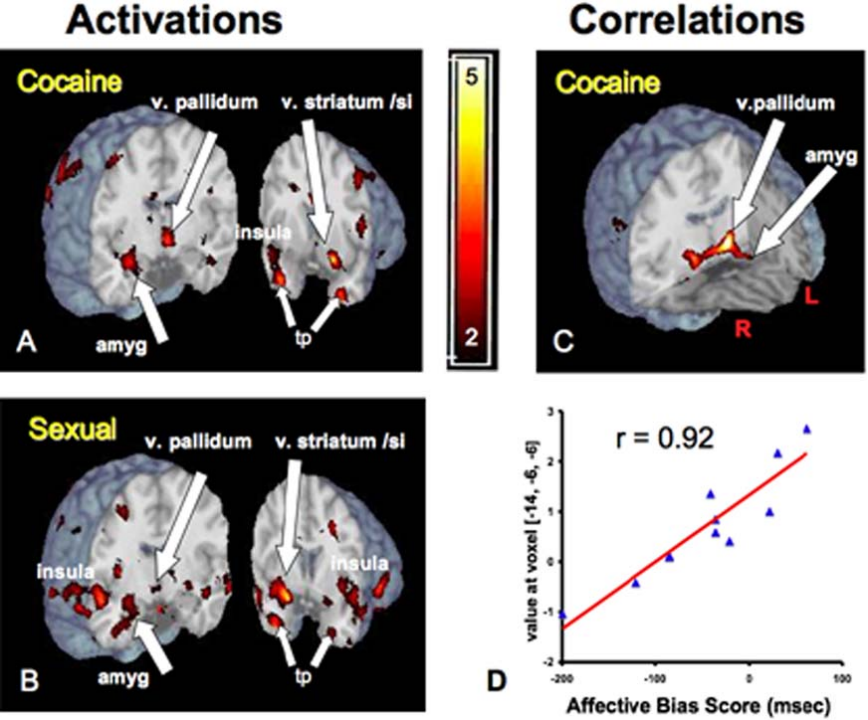
Limbic brain responses by 33 msec “unseen” cues in cocaine patients. Both cocaine **(A)** and sexual **(B)** cues produced activation in amygdala and ventral striatum/ventral pallidum/substantia innominata, and insula, as well as the OFC (OFC not shown). Statistical parametric t maps were generated by SPM 2; thresholded for display (color bar: 2<t<5) on the single-subject MNI brain template (“Colin”) in MRICro. Coronal brain sections on the left in (a) and (b) are at y = −6 mm; images on the right in **(A)** and **(B)** are at y = 10 mm and y = 6 mm, respectively. The response to “unseen” cocaine cues in a large bilateral ventral pallidum/left amygdala cluster **(C)** strongly predicted (peak voxel, MNI x, y, z coordinates: −14, −6, −6; t = 7.11; p = 0.000 uncorrected; p = 0.015 cluster-corrected) future affective response to visible cocaine cues **(D**; r = 0.92**)**. Brain response to 33 msec “unseen” aversive cues (*not shown; see* ***Figure S1*** *in Supporting Materials*) varied across individuals, with increased activity in the insula predicting the later affective response to visible aversive cues. Abbreviations: R: right, L: left, v: ventral, amyg: amygdala, si: substantia innominata, tp: temporal pole. [NOTE: The ventral boundary of the BOLD acquisition plane for the current studies is z = −40; temporal pole activations may extend ventrally below the acquisition plane.].

Strikingly, both the cocaine and sexual cues – though “unseen” as measured by the assessment tasks – produced differential activation of limbic brain regions. “Unseen” cocaine cues (Figure 2A; see supplemental Anatomical Comments and additional References in Text S1) activated the amygdala (a brain region rapidly assigning positive or negative hedonic valence to incoming stimuli), the ventral striatum and ventral pallidum (critical in reward processing), the insula (registering reports from the autonomic viscera/heart; linked both to emotional states and to addiction vulnerability), and the temporal poles, part of the classic limbic circuitry activated by extended cocaine cues [8]. “Unseen” cocaine cues also differentially activated caudal orbitofrontal cortex (important for weighing and updating the value of rewards (images not shown). “Unseen” sexual cues produced robust limbic activations, including the amygdala, ventral striatum/pallidum, orbitofrontal cortex, anterior insula, and temporal poles (Figure 2B), as well as posterior insula and the hypothalamus/midbrain tegmentum (latter images not shown). Overall, the brain's rapid response to drug and sexual cues presented outside awareness showed substantial anatomical overlap with its known response to longer cues that elicit conscious desire, with possible exception of higher frontal modulatory regions (see supplemental Anatomical Comments in Text S1).

### The brain response to “unseen” cocaine cues predicts future affect to visible versions of the same cues

By entering each individual's average affective bias score for cocaine cues, obtained from the off-magnet priming task (see Methods) into the relevant imaging contrast, we were able to test for correlations with rapid brain response to “unseen” cocaine cues. We found that greater brain activity to “unseen” cocaine cues in the interconnected ventral pallidum and amygdala predicted a more positive affective response to visible versions of the same stimuli in off-magnet testing two days later (Figure 2, C & D). This correlation underscores the functional significance of the brain's reaction to “unseen” reward cues, and suggests potential continuity between affective/motivational processes occurring within and outside awareness.

## Discussion

These findings highlight the exquisite sensitivity of the brain to signals for reward – and not only for rewards with clear survival significance (i.e., sexual opportunity [7]), but also for drug rewards with acquired, “as if” biologic significance because of their actions on the same brain circuitry. Though the amygdala is better known for its rapid response to signals for danger[2]; the response demonstrated here to “unseen” sexual and drug stimuli point to its importance in the processing of signals for reward[13]–[15], even when presented outside awareness. Further, though the role of the striatum/pallidum in reward processing has an extensive history (see supplemental Anatomical Comments and additional references in Text S1), this is the first evidence in humans for a striatal/pallidal response to “unseen” sexual and drug cues.

### Theoretical context

Our working hypothesis is that drug and sexual cues acquire the ability to trigger brain reward circuitry through simple Pavlovian conditioning. In Pavlov's classic experiments[16], a bell reliably signaling the arrival of food reward came to trigger salivation and excited anticipation of the meal. Though salivation to the food was reflexive, salivation and arousal to the bell was “conditional” – newly established by learning. Similarly, arbitrary cues (e.g., the sight of a drug paraphernalia, a drug location, or even an internal cocaine thought) that reliably signal cocaine will come to trigger physiologic arousal, drug anticipation, and activation of the limbic reward circuitry[8]in users of the drug. Because the cue-triggered responses depend on learning, cocaine-naïve individuals do not show these responses to cocaine cues [8], [17], [18].

In our view, the “hair-trigger” brain response of cocaine users to “unseen” drug and sexual cues reflects Pavlovian learning, but with a powerful evolutionary thrust. Organisms with a rapid response to reward signals – even brief or “unseen” signals – for food and sex would have a survival advantage. Cocaine confers no such advantage. However, because cocaine strongly activates the reward circuitry, the limbic brain treats cocaine cues “as if” they were signals for highly desirable natural rewards.

From this perspective, it is tempting to directly compare the magnitude of the brain response to cues for a “natural” (e.g., sexual) reward vs. an “unnatural” cocaine reward. An earlier study in cocaine users [18] indeed reported a stronger brain response to visible cocaine cues than to sexual cues, consistent with patient reports that cocaine desire is very similar to sexual desire, but much stronger. We did not directly compare the brain response to “unseen” sexual vs. cocaine cue categories because of the difficulty – for human cue-provocation studies – in equating the cues on a critical dimension: their relevance for the individuals' unique learning history. Even when the cue categories are otherwise roughly equated on perceptual features (brightness, hue, complexity) or normed on a reference population[19], the “fit” with prior conditioning history is expected to be a strong determinant of the brain response. As drug and sexual history are uncontrolled variables in human research, preclinical studies in the fMRI setting could offer a novel alternative for establishing, and then comparing, the brain response to “unseen” cues for drug vs. non-drug rewards.

### Limitations

As with any new finding, the generalizability and replicability of the current results with “unseen” cues will depend on additional studies. Encouragingly, in a very recent study, Pessiglione, et al.[20] have demonstrated similar (amygdala, striatum, pallidum) brain responses to cues for “unseen” monetary rewards, extending the generality of the current findings to a different reward and a different subject population. Studies with “unseen” cues for food, a critical natural reward, will be a valuable extension and validation of the current findings, as will studies with other clinical populations (e.g., those who struggle to manage motivation toward food or sexual reward).

### Clinical implications

The brain's rapid response to “unseen” reward cues may have clinical implications. The brain can strike up a prelude to passion in an instant, outside awareness, and without heavy policing from frontal regulatory regions. By the time the motivational state is experienced and labeled as conscious desire, the ancient limbic reward circuitry already has a running start. This dilemma may be reflected not only our daily human struggle to manage the pull of natural rewards such as food and sex, but also in the chronic, treatment resistant disorders for which poorly controlled desire is a cardinal feature (e.g., the addictions). Encouragingly, neuroimaging paradigms with “unseen” cues may be used to develop treatments that address problematic motivation at its earliest beginnings, i.e., outside awareness.

## Materials and Methods

### Subjects

#### Imaging cohort

Twenty-two treatment-seeking male cocaine patients (averages: 41.57 years of age; 15.68 years prior cocaine use) met the general medical and psychiatric inclusion/exclusion/stabilization criteria of our earlier PET O-15 study[8], with the additional fMRI exclusion criteria of metal in the body and known claustrophobia. The patients stayed in supervised residential setting (half-way house) for 7–10 days prior to scanning, to ensure a stabilized, drug-free state. The final eleven of these subjects (who did not differ demographically from the first half of the cohort), also participated in an off-magnet affective priming task two days after scanning, to determine the affective valence of the cocaine cues when visible.

#### Off-magnet cohort to test efficacy of the backward-masking paradigm

An additional 12 cocaine patients from the same residential treatment setting were matched for close demographic similarity (in age, years cocaine use, and years education; see Supplemental Methods *forced choice category recognition task*, below) to the imaging cohort. They participated in off-magnet testing to validate the backward-masking parameters used in the imaging setting.

#### Informed consent/ Human subjects approval

The reported procedures for human subjects were fully approved by the Office of Human Research (OHR) at the University of Pennsylvania. All subjects gave witnessed, informed (written) consent prior to participation.

### Procedures

#### Selection of stimuli

The 24 cocaine stimuli and the neutral stimuli were selected from our laboratory archive of drug and non-drug images. For the sexual and aversive stimuli, we selected (using male norms) the extremes of the affect-positive (“pleasant”) and affect-negative (“unpleasant”) images from the International Affective Picture Systems, IAPS[19]); replacing pictures of solitary nude males with erotic couples pictures from our archive. “Null” stimuli were simple grey screens with a small black fixation cross.

#### Presence of fear, anger, “gaze” and facial features in the selected study stimuli

As the amygdala can show preferential responding to several kinds of eliciting stimuli (e.g., anger[6], fear[21], threat[2], [5], “gaze” coupled with emotional expression[22] and even neutral faces[23]), we monitored our study stimuli for features that might complicate interpretation of the results for drug and sexual cues. The extreme-aversive IAPS stimuli featured severely injured, dead, or diseased people and animals, rather than direct facial expressions of anger or fear. Five of the aversive stimuli and five of the sexual stimuli involved a direct frontal gaze (though not angry or fearful); none of the cocaine stimuli involved a frontal gaze or angry/fearful expressions. Faces were represented in 52.4% of the aversive stimuli (though many faces were from individuals no longer alive), in 91.7% of the sexual stimuli (though faces were rarely the focus of the pictured activity), and 37.5% of the cocaine stimuli. Faces were not used in the neutral targets and masks; these stimuli featured simple household/office objects, outdoor objects, and outdoor scenes. 

#### Backward masking task

Following anatomical and localizer scans, subjects were instructed to view a series of brief picture stimuli. We used a “fast” event-related design[24], [25] to present, in randomized “jittered” [24]order, 24 unique pictures from the 4 categories of target stimuli: cocaine, sexual, aversive, and neutral, along with the interspersed “Nulls”. An Epson LCD computer projector used EPrime software to present the stimuli on a translucent white 28 1/2″×28 1/2″ screen (12″×20″ viewable) rear-projection screen positioned behind the scanner; the contents of this screen were reflected onto a 6 1/2″×3 1/2″ mirror positioned just above the subjects' eyes.

Activation contrasts were based on the first 120 stimulus presentations to minimize the contribution of habituation, recruitment, and recently documented “carry-over” effects that can occur with rapidly presented emotional stimuli (Yin Li, et al. Personal Communication of abstract presented at the College on Problems of Drug Dependence, Scottsdale, AZ, 2006); the presented correlational analyses were based on all 240 presentations.

#### Target duration in the backward-masking task

The target stimuli were 33 msec* in duration (*this actually varied between 33–34 msec, due to the refresh-rate of the computer screen). Each target was immediately followed by a neutral “mask” of 467–466 msec, to total 500 msec of visual stimuli.

In selecting 33 msec for target duration, we were guided by the range of stimulus durations in several earlier imaging studies demonstrating effective backward-masking of stimuli with affective valence. Effective visual masking has been shown with 33 msec targets of happy or fearful faces [4], [6], 30 msec targets of angry faces [5], [21] and 20 msec targets of happy or sad faces [26], [27], or combat/non-combat stimuli [28]. At target durations of 40 msec or longer, some subjects begin to visually recognize the targets (fearful or happy faces [29]; combat/non-combat stimuli [28], at levels above chance. As masking effectiveness depends not only on the duration of the targets, but can also depend on other variables (qualitative characteristics of both the targets and the masks, stimulus onset asynchrony, etc.), we also conducted explicit assessments (described next) to determine the effectiveness of our backward-masking parameters.

#### Assessing recall and recognition of the backward-masked targets

The kinds of recognition tasks used in “perception without awareness” paradigms have varied depending on the experimental context (whether the subject is to be aware or unaware of the target categories probed), goal of the experiments (whether focus is ‘object detection’ at the threshold of awareness, or full ‘object identification’), and theoretical interests of the investigators (see [30]–[34]).

In *immediate recall tasks*, subjects may be asked to describe what they have just seen during the immediately prior task, to pick out “seen” vs. “unseen” pictures amongst a set (as in Whalen, et al. 1998[6], or to designate individually presented items as previously “seen” or “unseen”. The addition of “Distracter” stimuli to a “seen vs. unseen” immediate recall task allows for an additional estimate of task engagement (distracters should be consistently rated as ‘unseen’, if the subject has been paying adequate attention), and possible detection of response-sets (e.g., endorsing a particular category of distracters or targets as “seen”).

Though responding at chance levels (or below) on an immediate recall task is consistent with the participants' subjective experience that the masked targets were indeed “unseen”, performance on the task is in fact dependent not only recognition, but also on potentially on memory (even if the recall task is only minutes later). At-chance or below-chance performance may reflect poor memory for the targets; the memory demands are even greater when the number of targets is large and the items within a category have some similarities (as in the current study).

To avoid the potential confound of memory demands, a *forced-choice category recognition task*, immediately following each individual target, can be used to probe immediate recognition of the stimulus. This task will be sensitive to any information that might help the subject differentiate the targets. Categorization of the target stimuli at- or below-chance levels – with the false positive rate taken into account – provides strong evidence that the target stimuli escaped recognition, and can therefore be considered “unseen” (outside awareness).

We employed both *immediate recall* and *forced choice category recognition* tasks to assess the efficacy of the backward masking parameters in our study. An *immediate recall* task was performed for each scanned subject, on-magnet. Immediately following the backward-masking task, subjects in the magnet were presented with a sequential series of 2.5 sec stimuli (5 from each target category, plus never-seen distracters) and asked to “Please indicate whether or not you have seen each picture (in the immediately preceding task).” This task probed a subset of five targets from each category, along with an equal number of distracter stimuli.

We also tested the recognition of our 33 msec backward-masked stimuli in a *forced-choice category recognition* task probing the entire set of 96 target stimuli (24 in each of 4 categories). The forced-choice category recognition task was conducted off-magnet, in a sample of twelve demographically-matched (see supplemental Results in Text S1) cocaine patients from the same residential setting as the scanned cohort. This off-magnet confirmation allowed us to conduct an independent, rigorous test for efficacy of our backward-masking procedure, without impacting the critical design feature of the imaging experiment – to determine the brain response to an uninterrupted flow of stimuli during natural, passive viewing, without the interruptions and potential confound of task demands/performance concerns. Both the targets and the backward-masks were the exactly the same (in number, duration and content) as in the magnet setting. The stimuli were presented on a (15″) Dell laptop computer screen positioned at desktop height, approximately 30 inches from the subject's head.

To introduce the task, subjects were told that their job was to categorize VERY BRIEF pictures immediately followed by pictures that would stay longer on the laptop screen. They were told the very brief pictures would be taken from 4 categories (neutral, sexual, cocaine, and disturbing), with equal numbers of pictures in each category. Verbal examples were given from each category (“….for example, disturbing pictures may include images of war, trauma, injury, sickness or death …”). Subjects were told they would have 2.5 seconds to make their choice, and should make their “Best Guess” in categorizing the very brief picture, even if they were not certain of the category. The 4 choice categories appeared as a row of 4 (large-text, boxed) words in the middle of screen following each backward-masked pair, and remained on the screen for 2.5 seconds or until a choice was made. Subjects made their selection by pushing one of 4 buttons on a response box; each button was clearly labeled with one of the four target categories. After two practice trials (for familiarity with the button box), the entire series of 96 targets, backward-masked, were presented for categorization. Following completion of the task, subjects were also debriefed about their subjective experience during the task, including whether they had “seen” one or more of the brief targets.

#### Affective priming task (performed off-magnet, 2 days after the on-magnet backward-masking task)

This task, adapted from Fazio [35], determined the affective valence of our targets by measuring their ability to facilitate (as measured by reaction time, RT), or “prime” the correct identification of affect-congruent nouns presented immediately after the target. Primes with a positive affective valence facilitate the correct identification of nouns with a positive valence (e.g., joy, paradise), and slow (retard) correct identification of nouns with a negative (e.g., vomit, murder) valence. Primes with a negative affective valence have the converse effect.

For each subject, a quantitative “affective-bias” score reflecting valence was calculated for each picture stimulus category, filtered for correct responses: (meanRT(NEGword-trials)-meanRT(POSword-trials)) = Mean Affective-Bias score for the stimulus category. With this formula, positive reaction time scores (msec) reflect relatively greater positive affect bias; negative reaction time scores reflect a relatively greater negative affective bias. Pictures for the off-magnet affective bias task were a randomly selected subset of those used in the fMRI experiment; 12 pictures from each category were used, for a total of 48 primes.

### Imaging acquisition and analysis

#### Image acquisition parameters

A Siemens 3T scanner was used for acquiring T2*-weighted Blood Oxygen-Level-Dependent (BOLD) images with single shot gradient echo (GRE) echo planar imaging (EPI) sequence (field of view (FOV) = 192 mm, matrix 64×64, TR = 2 sec, TE = 30 msec, flip angle = 80°.

#### Image pre-processing and statistical analysis

After slice-timing correction of the images, SPM2 [36]was used for image realignment, smoothing with a 3-D 9 mm isotropic Gaussian kernel, and normalization into the Montreal Neurological Institute averaged template based on structural MRIs from 152 brains. The General Linear Model with a canonical HRF as the basis function was used for three pre-planned contrasts (cocaine vs. neutral, sex. vs. neutral, aversive vs. neutral) at the individual and group level. For the correlational analysis, affective bias scores from the off-magnet affective priming task were used as covariates in a simple regression (e.g., cocaine vs. neutral contrast). Contrasts and correlations were displayed within MRICro, using the MNI single-subject t1 structural MRI brain template (the “Colin” brain template, closest to the average of the MNI 152 template; “Colin's” brain was scanned multiple times by MNI to result in a crisp visual display.

#### Analytic approach and thresholding of the statistical parametric maps

Because of the proximity and highly interconnected nature of several nodes of the reward system (see supplemental Anatomical Comments, following supplemental Results, in Text S1) – and the usual limitation on spatial resolution associated with warping of data into a standard 3-D space – we chose a voxel-based analysis, allowing us to characterize effects that might occur at the boundary of two interconnected *a priori* regions (e.g., amygdala and ventral striatum/pallidum).

We used the large number of prior published articles describing the interconnected circuitry activated by reward cues [12] (see supplemental Anatomical Comments in Text S1) to limit our hypotheses and interpretations to regions of strong a priori interest. We thresholded the statistical maps at t = 2.0 (height), k = 10 contiguous voxels (extent) both for the basic “group effect” contrasts (e.g., cocaine cues vs. neutral cues) and for the correlational analyses to examine the significance of individual variation in the brain response to “unseen” cues. The calculated p values (<0.029 and 0.038, respectively) associated with this selected threshold are somewhat more stringent than, but in the range of, the p<0.05 uncorrected threshold used in imaging studies with strong *a priori* regions of interest. It is worth noting that peak t-values, for especially for the correlational analyses, greatly exceeded the t = 2.0 threshold: As shown in Figure 2, C and D, individual differences in the brain response to the “unseen” cocaine cues (pallidal/amygdala cluster) predicted later affect to visible versions of these cues with peak t value = 7.11; p<0.000 uncorrected; p <0.015 cluster-corrected.

Peak t values for the correlational effects to “unseen” cocaine cues were generally higher than peak values for “group” activation effects, underscoring the strong individual variation in the brain response to “unseen” cues. Individual variation was also prominent in the response to the aversive comparison cues (see Figure S1, in Supporting Materials). A powerful feature of our paradigm was the incorporation of affective bias scores as regressors for the imaging analyses, allowing us to test for the functional significance of individual variability in the response to “unseen” cues.

## Supporting Information

Text S1 Supporting Information, including additional detail for Methods, Results, and additional detail for Discussion (including Anatomical Comments), associated References, and Figure Legend for Figure S1
(0.12 MB DOC)

Table S1 Tabled data from Forced-choice categorization recognition task, showing target stimuli are not recognized at the study parameters.
(0.04 MB DOC)

Figure S1 Though there was no significant overall group effect for “unseen” aversive IAPS vs. neutral stimuli in this cohort (A), individual variation in brain response in the insula (B) was strongly predictive of future affective response to visible versions of these stimuli, as illustrated for voxel [-54,-8,-12] of left insula (C).
(0.13 MB TIFF)
