# Supplementary material for: Prelude to Passion: Limbic Activation by “Unseen” Drug and Sexual Cues
Source: PLoS One. 2008 Jan 30;3(1):e1506. doi: 10.1371/journal.pone.0001506 (PMC2204052; doi:10.1371/journal.pone.0001506)
Supplement: Text S1 — Supporting Information, including additional detail for Methods, Results, and additional detail for Discussion (including Anatomical Comments), associated References, and Figure Legend for Figure S1 (0.12 MB DOC) [file pone.0001506.s001.doc]

Supporting Text

## Title

Prelude to Passion: Limbic Activation by “Unseen” Drug and Sexual Cues

## Authors and Affiliations

Anna Rose Childress^1, 3^, Ronald N. Ehrman^1, 3^, Ze Wang^1,2,3^, Yin Li ^1,2,3^, Nathan Sciortino^1^, Jonathan Hakun^1^,William Jens^1^, Jesse Suh^1, 3^, John Listerud^1, 3^, Kathleen Marquez^1,3^, Teresa Franklin^1^, Daniel Langleben^1, 3^, John Detre^2^ and Charles P. O’Brien^1, 3^

University of Pennsylvania School of Medicine, Department of Psychiatry^1^ and Neurology^2^, and VA VISN 4 MIRECC, Philadelphia Department of Veteran’s Affairs Medical Center^3^.

Corresponding Author: Anna Rose Childress, Ph.D.

e-mail: [childress_a@mail.trc.upenn.edu](mailto:childress_a@mail.trc.upenn.edu)

Center Phone: 1-215-222-3200 (Press x100 and ask receptionst to page Dr. Childress)

Cell Phone: 1-610-764-0038

FAX: 1-215-386-6770

_____________________________________________________

**Supplemental Results**

*Recall and Recognition tasks* (testing efficacy of the backward-masking procedure).

*“Seen vs. unseen” Immediate Recall task*. All of the probed targets [and distracter stimuli] were endorsed as “previously seen” at levels (range: 0.47 to 1.40) well below the 2.5 (out of 5 probes) expected by chance. The neutral masking stimuli (as expected) were recalled as “previously seen” at levels above chance (average: 3.67 of the 5 test probes). This pattern of results in the “seen vs. unseen” immediate recall task is consistent both with effective backward masking and with appropriate task engagement (above-chance recall of the Masking stimuli and low “false positive” recall for the Distracter stimuli).

*Forced-choice Category Recognition task:* Data from the button response box failed to be encoded by the computer for two of the twelve selected cocaine patients. The ten cocaine patients with recorded task data remained demographically well-matched to the on-magnet sample, not differing significantly (two-tailed t-tests for independent samples, 0.31 < p < 0.87) in mean age (41.95 vs. 41.58 years), years of cocaine use (14.73 vs. 15.68 years), or years of education (11.88 vs. 12.45).

*Objective data from the Forced-choice Category Recognition task:* As shown in **Table S1**, below, the forced-choice category task also yielded results consistent with effective backward-masking of the targets. With four target categories available (the masks were visible neutral pictures), an average of 25% button presses or less for each target category represented a chance level of endorsement for the category. Cocaine (10%), Aversive (12%), and Sexual (25%) categories were endorsed at or below chance levels. Only

the Neutral category (targets and masks were from different pools of neutral pictures) was endorsed at a level above chance (53%). Very importantly, the “above-chance” endorsement of the neutral category did not reflect a better rate of *accurate* *neutral target* categorizations, as the ratio of “false positive” endorsement for targets was high for all four categories (see **Table S1**), including the neutral category. Pair-wise comparisons of false positive ratios (for patients having at least one false positive and one true positive endorsement for each category) showed there was no statistically significant difference in false positive ratio among the four categories (one-way ANOVA with repeated measures, followed by pair-wise comparisons; all p > 0.48). Over-endorsement of the neutral category thus likely reflects a simple default categorization, as the 467 msec neutral masks were visible and easy to see, while the 33 msec targets were not (a regular comment of the subjects at de-briefing).

*Subjective data from the Forced-choice Category Recognition task:* When asked to estimate their overall performance, 8 of 10 patients felt bad or uncertain about their performance (e.g., “Bad”, “Don’t think I did too well”, “Not too good; too fast”, “Hard to say…”). One of the two remaining patients felt he was “great on the sexual ones”, but his false positive rate for the sexual category was the highest of all the subjects tested. (At the end of the task, without prompting, he asked if he might speak to a clinician about his strong sexual impulses). The other subject felt very optimistic about his overall performance “75% right”, but his actual performance pattern showed that he endorsed the neutral target category for 72 % of his presses.

When asked for any specific examples of “identifiable” targets (from among the 96 backward-masked stimuli shown to each subject), spontaneous identification rates were extremely low. Each male subject felt he may have “seen” at least one target with sexual content (e.g.,“naked…girl”, “couple”), with one subject (described previously) feeling that he may have “seen” much sexual material (his high rate of false positive categorizations would suggest otherwise). Three subjects felt they had “seen” a single drug-related target, but one of these drug targets was described as a “syringe” (there were no syringe pictures among the targets, as the cocaine cues were all related to smoked crack cocaine). Only one aversive target (“a disturbing internal organ?”) was mentioned, by a single subject.

The results from both the *immediate recall* task and the *forced-choice category recognition* task support the effectiveness of the backward masking parameters used in the current study. Sample target stimuli were not recalled as “seen” when probed immediately following the scan, and independent testing of all 96 targets in a rigorous forced-choice category task showed they were not accurately recognized under the current study parameters (33 msec targets; 467 msec backward-masks).

*Brain activations and correlations for “unseen” 33 msec aversive stimuli*. Though the brain response for “unseen” **cocaine** cues evidenced both shared “group” effects *and* meaningful inter-subject variability, it is worth noting that meaningful inter-subject variability can exist in the absence of a significant “group” effect. This is dramatically illustrated by the brain response to “unseen” **aversive** cues in the current paradigm.

There was no overall group effect (**Figure S1, A** -- below) for the extreme-aversive IAPS stimuli under the current backward-masking parameters. As longer presentations of aversive (and appetitive) IAPS stimuli [1,2] do produce clear group effects in the brain, we hypothesized that the early brain response to the 33 msec aversive IAPS stimuli (e.g., grossly disfigured or injured faces, decomposing or charred animal bodies, etc.) might have functionally significant inter-subject variability, i.e., that subjects would vary in their sensitivity to “unseen” disturbing stimuli. To test this possibility directly, we used each individual’s off-magnet affective bias score for visible aversive stimuli (as described above in **Supplemental Procedures** for Affective Bias task) as a covariate of interest (simple regression) in the aversive vs. neutral contrast. This correlational approach revealed that brain activity in the insula (bilaterally) to “unseen” 33 msec aversive targets strongly predicted the

future (two days later) negative affective bias to visible versions of these stimuli

(**Figure S1, B**). A robust predictive response for the left posterior insula is plotted in **Figure S1, B** (r = - 0.87 for voxel [-54, -8, -12], one of several local maxima ( t = 5.3) in a large predictive cluster that extends posteriorly for several mm (k extent = 10 voxels; p < 0.000, cluster-corrected). The right anterior insula was also predictive (r = - 0.85) of future negative affect, but with peak t values (t = 4.81 [54, 20, -10]) of a lower cluster-corrected p value ( p < 0.023).

These results are consistent with the role of the insula in interoceptive states [3], including aversion [4], and provide the first evidence, to our knowledge, that the insula response to “unseen” aversive cues predicts future negative affect to visible versions of these cues. The results for aversive “unseen” cues, though not the focus of the current study, underscore the importance of inter-subject variability in the brain response to evocative cues[5], and the ability of appropriate regressors, such as the affective bias scores, to reveal the functional significance of the varying brain response. The different pattern of brain response to the “unseen” aversive vs. the appetitive (cocaine and sexual) cues supports the specificity of our selected probes for the cohort we studied, and more generally, the specificity of the brain response to stimuli of negative vs. positive valence, even when “unseen”. The current results also suggest the “unseen” aversive IAPS cues may be useful imaging probes in populations with clinical problems (e.g., anxiety, post-traumatic stress disorder, contagion fear, depression) related to the processing of negative affect.

**Supplemental Discussion**

**Supplemental Anatomical Comments**

*Extended amygdala and striatal/pallidal system.* The activations to "unseen" 33 msec reward cues occurred both in the amygdala proper, and in a region that encompassed the ventrolateral striatum, lateral pallidum, and the substantia innominata. In Figure 2 a and b, we labeled the posterior extent of this functional activation as ventral pallidum, and the more anterior extent as the ventral striatum/substantia innominata.

The striking similarities in cellular architectonics, anatomical connections and neutrotransmitters between the amygdala and the ventral striatum/nucleus accumbens have led Heimer [6] to propose these structures as part of an “extended amygdala”(SLEA, sublenticular extended amygdala). In Heimer’s view, the extended amygdala, together with the ventral striatal-pallidal system and the adjacent substantia innominata (also know as the basal nucleus of Meynert) are the primary output channels for the limbic lobe’s critical motivational and emotional functions[6]. The current results show that reward cues as brief as 33 msec can activate the SLEA, and that awareness is not required. The critical importance of the striatum/pallidum in reward processing has extensive support from preclinical research [7-15], the current imaging results affirm their importance in rapid human reward processing that does not require awareness.

It is of potential interest that activations in the amygdala proper, for “unseen” cocaine and sexual cues, appeared to be in the lateral subregion. Given the limited spatial resolution of fMRI (3mm) relative to the size of the amygdala (8mm at maxima), any conclusions regarding amygdala subregions are necessarily tentative. However, if replicable in future similar studies, activation of the lateral amygdala subregion would be consistent with elegant preclinical studies pointing to the role of the basolateral amygdala in assigning affective valence to Pavlovian appetitive signals [16].

*Insula*. The insula is interconnected with the amygdala, and receives multi-modal sensory and visceral inputs. The integration of these inputs creates interoception[3], the organism’s sense of its physiological condition. The learned response of the insula to cocaine reward cues may account for several drug-like phenomena of cue-induced cocaine craving that occur prior to actual ingestion of the drug: e.g., “stomach flipping”, “heart pounding”, the “taste” of cocaine in the back of the user’s throat, and the “smell” of cocaine in an otherwise empty room, and brief euphoria. These internal drug-like responses are often conscious, and are described by users as having incentive properties – pulling the user toward the full drug reward. However, as illustrated by the current study, rapid activation of the insula to appetitive cues does not require recognition of the triggering stimuli. Insula activation by “unseen” cues may be a precursor to consciously-experienced drug craving, perhaps explaining why insula damage in smokers was recently found to be associated with a dramatic loss of smoking urges[17].

*Orbitofrontal cortex (OFC).* The OFC is closely interconnected with the amygdala, and is responsible for evaluating and updating the hedonic value of rewards (or punishments) based upon this communication. Though not highlighted in the 3-D cut-away views of Figure 2, the caudal aspect of the right OFC was differentially activated by “unseen” cocaine cues; for “unseen” sexual cues, activation included not only the caudal OFC bilaterally, but also the most medial aspect of the ventral OFC, the gryus rectus. As described in the elegant meta-analysis of Kringelbach and Rolls[18], reward-related processes are localized in the medial OFC, with the less abstract rewards represented in a more caudal direction. The pattern of OFC response to 33 msec “unseen” reward signals is consistent with these anatomical specializations. The caudal OFC activation by “unseen” cocaine cues additionally suggests that cocaine cues may be experienced as more similar to primary rewards (such as sex) than to abstract rewards (such as money).

*Frontal cortical regions.* Activation of the supragenual anterior cingulate and medial prefrontal cortex is a common response to longer cues for natural rewards and for drugs of abuse, perhaps reflecting focused attention, and/or attempts (reflexive or voluntary) to modulate the response. However, higher cortical activations were not prominent in the brain response to 33 msec cues. The response of higher cortical regions likely takes several milliseconds longer to recruit than the amygdala and other ‘first responder’ systems.

In support of this interpretation, elegant studies using optimized magnetoencephalo-graphy (MEG)[19] have recently shown that the brain response to motivationally significant fear stimuli was earliest in the hypothalamus (10-20 msec), followed by the amygdala (20-30 msec), thalamus, basal ganglia, insula, and then the inferior frontal gyrus. The response from the frontal regions occurred relatively slowly, on a time scale of 160 to 210 msec, consistent with the role of these regions in integrating multiple inputs and providing modulatory feedback.

Though MEG studies of this type have not yet been conducted with ultra-brief reward-related stimuli, our detection of a differential response to 33 msec cocaine and sexual cues in the amygdala, striatum, and insula suggests these systems are responding as rapidly to reward cues as has been historically demonstrated for fear cues. In addition, the general lack of higher cortical activations suggests that brief “unseen” cues -- though having a clear subcortical impact -- may receive less frontal regulation. Motivation that is stirred outside awareness and outside frontal regulatory processes may be particularly difficult to manage, especially for individuals with psychopathologies that include compromised frontal modulatory function (e.g., addictions, post-traumatic stress disorder, affective disorder, and attention-deficit-hyperactivity disorder). These individuals may benefit from treatments directly targeting subcortical elements of the brain response to “unseen” cues.

**_______________________________________________________________________**

**References for Supporting Text**

1. Garavan H, Pendergrass JC, Ross TJ, Stein EA, Risinger RC (2001) Amygdala response to both positively and negatively valenced stimuli. Neuroreport 12: 2779-2783.

2. Hamann SB, Ely TD, Hoffman JM, Kilts CD (2002) Ecstasy and agony: activation of the human amygdala in positive and negative emotion. Psychol Sci 13: 135-141.

3. Craig AD (2003) Interoception: the sense of the physiological condition of the body. Curr Opin Neurobiol 13: 500-505.

4. Nitschke JB, Sarinopoulos I, Mackiewicz KL, Schaefer HS, Davidson RJ (2006) Functional neuroanatomy of aversion and its anticipation. Neuroimage 29: 106-116.

5. Canli T, Sivers H, Whitfield SL, Gotlib IH, Gabrieli JD (2002) Amygdala response to happy faces as a function of extraversion. Science 296: 2191.

6. Heimer L (2003) A new anatomical framework for neuropsychiatric disorders and drug abuse. Am J Psychiatry 160: 1726-1739.

7. Tindell AJ, Smith KS, Pecina S, Berridge KC, Aldridge JW (2006) Ventral pallidum firing codes hedonic reward: when a bad taste turns good. J Neurophysiol 96: 2399-2409.

8. Wise RA (2005) Forebrain substrates of reward and motivation. J Comp Neurol 493: 115-121.

9. Tang XC, McFarland K, Cagle S, Kalivas PW (2005) Cocaine-induced reinstatement requires endogenous stimulation of mu-opioid receptors in the ventral pallidum. J Neurosci 25: 4512-4520.

10. Galvan A, Hare TA, Davidson M, Spicer J, Glover G, et al. (2005) The role of ventral frontostriatal circuitry in reward-based learning in humans. J Neurosci 25: 8650-8656.

11. Kelley AE (2004) Ventral striatal control of appetitive motivation: role in ingestive behavior and reward-related learning. Neurosci Biobehav Rev 27: 765-776.

12. Robbins TW, Everitt BJ (2002) Limbic-striatal memory systems and drug addiction. Neurobiol Learn Mem 78: 625-636.

13. Koob GF (1999) The role of the striatopallidal and extended amygdala systems in drug addiction. Ann N Y Acad Sci 877: 445-460.

14. Roberts DC, Corcoran ME, Fibiger HC (1977) On the role of ascending catecholaminergic systems in intravenous self-administration of cocaine. Pharmacol Biochem Behav 6: 615-620.

15. Rademacher DJ, Kovacs B, Shen F, Napier TC, Meredith GE (2006) The neural substrates of amphetamine conditioned place preference: implications for the formation of conditioned stimulus-reward associations. Eur J Neurosci 24: 2089-2097.

16. Everitt BJ, Cardinal RN, Parkinson JA, Robbins TW (2003) Appetitive behavior: impact of amygdala-dependent mechanisms of emotional learning. Ann N Y Acad Sci 985: 233-250.

17. Naqvi NH, Rudrauf D, Damasio H, Bechara A (2007) Damage to the insula disrupts addiction to cigarette smoking. Science 315: 531-534.

18. Kringelbach ML, Rolls ET (2004) The functional neuroanatomy of the human orbitofrontal cortex: evidence from neuroimaging and neuropsychology. Prog Neurobiol 72: 341-372.

19. Luo Q, Holroyd T, Jones M, Hendler T, Blair J (2007) Neural dynamics for facial threat processing as revealed by gamma band synchronization using MEG. Neuroimage 34: 839-847.

___________________________________________________________________________

**Figure Legend for Supporting Figure S1**

**Figure S1.** Though there was no significant overall group effect for “unseen” aversive IAPS vs. neutral stimuli in this cohort **(A),** individual variation in brain response in the insula **(B)** was strongly predictive of future affective response to visible versions of these stimuli, as illustrated for voxel [-54,-8,-12] of left insula **(C).**
