# Supplementary material for: Prelude to Passion: Limbic Activation by “Unseen” Drug and Sexual Cues
Source: PLoS One. 2008 Jan 30;3(1):e1506. doi: 10.1371/journal.pone.0001506 (PMC2204052; doi:10.1371/journal.pone.0001506)
Supplement: Table S1 — Tabled data from Forced-choice categorization recognition task, showing target stimuli are not recognized at the study parameters. (0.04 MB DOC) [file pone.0001506.s002.doc]

**Table S1**

Categorization of backward-masked targets in the Forced - Choice Category Recognition (“Best Guess”) Task.

| **Category** | **Mean Percent (+ s.d.)*** | **Mean False Positive Ratio*** |
| --- | --- | --- |
|  | (of total presses) | (false positive / "true" positive) |
| **Neutral** | **53** (+29) | **1.94** |
| **Aversive** | **12** (+12) | **1.84** |
| **Sexual** | **25** (+13) | **1.96** |
| **Cocaine** | **10** (+09) | **1.75** |

* Mean percent presses were based on all 10 subjects. False positive ratios were calculable for individuals having (at minimum) one false positive and one “true” positive endorsement per category (Neutral, n=10; Sexual, n=10, Aversive, n=8; Cocaine, n=7). The 7 subjects with calculable False Positive ratios for all 4 categories were used for subsequent pair-wise comparisons of False Positive Ratios.
