# Supplementary figures and images for: Prelude to Passion: Limbic Activation by “Unseen” Drug and Sexual Cues
Source: PLoS One. 2008 Jan 30;3(1):e1506. doi: 10.1371/journal.pone.0001506 (PMC2204052; doi:10.1371/journal.pone.0001506)

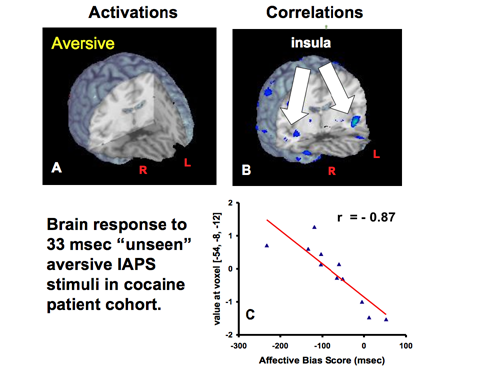

Supplement: Figure S1 — Though there was no significant overall group effect for “unseen” aversive IAPS vs. neutral stimuli in this cohort (A), individual variation in brain response in the insula (B) was strongly predictive of future affective response to visible versions of these stimuli, as illustrated for voxel [-54,-8,-12] of left insula (C). (0.13 MB TIFF) [file pone.0001506.s003.tif]
